# First 100 patients receiving long-acting growth hormone therapy: real-world evaluation from INSIGHTS-GHT registry

**DOI:** 10.1186/s13023-025-03898-8

**Published:** 2025-07-23

**Authors:** Joachim Woelfle, I. Kreitschmann-Andermahr, C. J. Strasburger, D. B. Pittrow, C. Pausch, D. Schnabel

**Affiliations:** 1 Department of Pediatrics and Adolescent Medicine, University Hospital Erlangen, Loschgestr. 15, Erlangen, Germany; 2 Department of Neurosurgery and Spine Surgery, University Hospital Essen, University of Duisburg-Essen, Essen, Germany; 3 Department of Endocrinology and Metabolism, Charité - Universitätsmedizin Berlin, corporate member of Freie Universität Berlin, Humboldt-Universität zu Berlin, Berlin Institute of Health, Berlin, Germany; 4 Institute for Clinical Pharmacology, Technical University, Dresden, Germany; 5 Innovation Centre Real-World Evidence, GWT-TUD, Dresden, Deutschland; 6 Pediatric Endocrinology / Center for chronic sick children, Charité, University Medicine, Berlin, Germany

**Keywords:** Pediatrics, Growth hormone, Lonapegsomatropin, Somapacitan, Somatrogon, Non-interventional study

## Abstract

The development of long-acting growth hormone (LAGH) formulations offers a promising approach to reduce injection frequency and to improve adherence in growth hormone deficiency (GHD) treatment. INSIGHTS-GHT is the first product-independent registry to document the real-world use of recombinant human (rh) growth hormone (GH) replacement therapy within the labelling. Following the market launch of three LAGH products in Germany (lonapegsomatropin, somapacitan, and somatrogon) we aimed to provide early real-world evidence on their use in order to obtain an initial picture on patient selection and physician preferences outside of clinical trials.

We report in this interim analysis on 70 pediatric patients from 15 centers across Germany as well as 31 adult patients from 6 German centers under LAGH treatment. The majority of the pediatric patients (76%) were male, with a mean age at LAGH initiation of 9.2 years. About half of the pediatric patients (54%) were switch patients transitioning from daily GH therapy. Notably, 82% of patients received a LAGH starting dose below the manufacturer’s recommendation, with a median dose of 92% of the recommended level. In the group of adult patients, 65% were male, with a mean age of 38.2 years at LAGH initiation. In pediatric patients, before start of GH therapy, mean IGF-I (SDS) was − 2.1 ± 1.1 SDS, mean IGFBP-3 (SDS) was − 2.0 ± 1.5 SDS.

All adult patients switched from daily GH therapy. More than half (55%) received the LAGH starting dose according to the manufacturer’s recommendation, while 41% began with a lower-than-recommended dose. Our findings provide early insights into LAGH therapy adoption and highlight the need for continued follow-up to evaluate long-term efficacy, adherence, and safety in real-world settings.

## Background

Over the past two decades, pharmacological research on growth hormone (GH) has increasingly focused on the development of long-acting growth hormone (LAGH) formulations to extend the duration of action of GH and reduce the frequency of injections required. These innovations have the potential to reduce treatment burden, enhance patient adherence, and improve clinical outcomes, particularly for individuals struggling with daily GH injections. Additionally, the integration of LAGH into clinical practice for pediatric patients could positively impact on both quality of life and cost-effectiveness [1]. 

Currently, three long-acting LAGH products are available in Germany, each with distinct pharmacological properties:

- Lonapegsomatropin (Skytrofa^®^) is a prodrug in which native GH is transiently bound to a methoxypolyethylene glycol carrier via a cleavable linker. Upon subcutaneous administration, enzymatic cleavage releases the active hormone, enabling sustained action [2].
- Somapacitan (Sogroya^®^) achieves prolonged activity through non-covalent, reversible albumin binding. By temporarily attaching to albumin in the bloodstream, its degradation is delayed, effectively extending its half-life [3].
- Somatrogon (Ngenla^®^) is a fusion protein combining native GH with three copies of the carboxy-terminal peptide from human chorionic gonadotropin. This structural modification increases molecular size, delaying renal clearance and enhancing its duration of action [4].

All three LAGH products are administered subcutaneously once weekly. Each agent induces a steady increase in insulin-like growth factor I (IGF-I) levels, with peak concentrations occurring approximately two to three days post-injection and maintaining therapeutic levels throughout the dosing interval.

The indications of the 3 products differ: Lonapegsomatropin and somatrogon are approved for the treatment of pediatric growth hormone deficiency (GHD) (children 3 to < 18 years), whereas somapacitan is approved for both, pediatric and adult, GHD patients, allowing for continuous therapy without the need for a treatment switch during the transition to adulthood.

Although estimates on the prevalence of GHD vary from 1:9000 up to 1:1000 in children [5] and from 0.2 to 37:100.000 in adults [6], GHD is not uniformly rare. Certain subtypes such as organic GHD from congenital anomalies or syndromic causes fall within the spectrum of rare endocrine disorders. Furthermore, diagnostic criteria for GHD differ between countries, including varying IGF-I cutoff-concentrations from 0 to -2 SDS [7]. Data from INSIGHTS-GHT may therefore support evidence-based use of GH in these less common presentations, for which data from randomized controlled trials are often absent.

To date, there is still limited experience with the therapeutic use of LAGH products. A first consensus statement on the use of LAGH in pediatric GHD was recently published [8]. To add to the initial real-world experience with LAGH, this report presents the first interim evaluation of patients with GHD receiving LAGH therapy, based on a database analysis on February 27, 2025.

## Methods

INSIGHTS-GHT is the world’s first cross-product registry study on GH therapy [9]. It includes patients of all ages and across all approved indications in Germany, allowing documentation of real-world GH treatment patterns from early childhood to adulthood, thus enabling to address numerous relevant scientific questions.

The study is conducted in specialized pediatric and adult endocrinology centers across Germany. It includes patients of any age who are receiving ongoing or newly initiated treatment with any approved somatropin product. The inclusion criteria are based on the approved indications of each somatropin product, ensuring that all disease entities explicitly mentioned in the regulatory label (e.g., pediatric and adult growth hormone deficiency, Turner syndrome, chronic renal insufficiency, and others as per product approval) can be documented comprehensively. Patients are treated as per standard (local) clinical practice.

Safety assessments in INSIGHTS-GHT encompass the documentation of all adverse events (both overall and those deemed causally related and/or temporally associated). The study aims to provide comprehensive real-world evidence on various aspects of somatropin treatment, including patient characteristics, treatment patterns, physician preferences and outcomes, regardless of the specific product used. Data are documented online in an electronic data capture system, undergo thorough quality assessment including plausibility checks on entry, statistical checks on outlier data, and onsite monitoring of participating sites.

Since the launch of the registry in February 2022, more than 2100 patients from 30 endocrinological institutions in Germany have been enrolled.

Baseline laboratory assessments include measurements of IGF-I and IGFBP-3 concentrations, which are documented as both absolute values and standard deviation scores (SDS) to facilitate standardized interpretation across age and sex-adjusted norms.

## Results

### Pediatric patients

By now, 70 children and adolescents (< 18 years) with GHD receiving LAGH therapy from 15 centers have been included. Of these, 76% are male. Idiopathic GHD was diagnosed in 70% of the patients, while 30% had organic GHD. The mean age at the start of LAGH therapy was 9.2 ± 3.8 years, with 79% of patients being prepubertal at the start of treatment. A total of 38 patients of 68 patients with known therapy history (56%) had previously been treated with daily GH injections for an average of 4.1 ± 2.8 years (‘switch patients’). At the initiation of LAGH therapy, the mean height SDS was -1.8 ± 1.4, the mean BMI SDS was − 0.4 ± 1.2. Before start of GH therapy, in pediatric patients mean IGF-I (SDS) was − 2.1 ± 1.1 SDS, mean IGFBP-3 (SDS) was − 2.0 ± 1.5 SDS. However, since this register study represents real-life data reported from numerous participating institutions, using several different IGF-I and IGFBP-3 assays, SDS calculations were only available in 37% and 39% while absolute serum concentrations were available in 77% and 71% of reported patients for IGF-I and IGFBP-3, respectively.

Further details, including data by indication and GH-naïve patients, are summarized in Table 1. Notably, the starting dose of LAGH was below the manufacturers’ recommendation in 83% of cases, with a median of 92% of the recommended level. Two adverse events under LAGH therapy were reported in two patients: headache and epistaxis.

**Table 1:** Characteristics of pediatric LAGH patients

Patient characteristics	Pediatric GHD Patients with LAGH Therapy (*n* = 70)	“Switch” Patients (*n* = 38)		LAGH First Line Prescription (*n* = 30)
Age at Diagnosis (years)	6.4 (3.6)	5.9 (3.5)		7.2 (3.8)
Age at initiation of rhGH/LAGH Therapy (years)	7.1 (3.6)	6.4 (3.4)		7.9 (3.6)
Diagnosis to GH Therapy Start (Median, months)	2.1	0.8		2.9
Age at Start of LAGH Therapy (years, range)	9.2 (3.8) (2.9–16.7)	10.5 (3.5) (2.9–16.7)		7.9 (3.6) (3.1–16.0)
Prepubertal at Start of LAGH Therapy (%)	78.8	70.3		88.9
Gender (Male/Female, %)	75.7 / 24.3	68.4 / 31.6		83.3 / 16.7
Idiopathic/Organic GHD (%)	70.0 / 30.0	60.5 / 39.5		80.0 / 20.0
Duration of Prior Treatment (years)	-	4.1 (2.8)		-
Height at Start of LAGH Therapy (SDS)	-1.8 (1.4)	-1.2 (1.0)		-2.3 (1.6)
BMI at Start of LAGH Therapy (SDS)	-0.4 (1.2)	-0.5 (1.1)		-0.3 (1.2)
Starting Dose (mg/kg per week)				
Somatrogon (Ngenla^®^)	0.59 (0.10)			
Lonapegsomatropin (Skytrofa^®^)	0.21 (0.04)			
Somapacitan (Sogroya^®^)	0.14 (0.03)			
Starting Dose below recommended dose (% of patients) across all LAGH	82.5	83.3		81.5
Percentage Starting Dose to recommended dose in prescribing information				
(Mean (SD),	87.6 (15.9),	89.0 (15.6),		85.7 (16.4),
Median)	91.7	93.8		87.5
IGF-I before start of GH therapy, ng/ml = ug/l	62.8 (56.2)		56.9 (42.8)	76.1 (71.8)
, SDS	-2.1 (1.1)		-2.2 (0.9)	-1.8 (1.8)
IGFBP-3 before start of GH therapy, ng/ml = ug/l	2031 (1080)		1922 (1059)	2256 (1136)
, SDS	-2.0 (1.5)		-2.3 (1.2)	-1.1 (2.2)

Values are means (SD), if not otherwise mentioned

### Adult patients

Currently, 31 adult patients from six centers are receiving somapacitan, 65% of whom are male. The majority (84%) have organic GHD, and in 57% of the cases, the condition was diagnosed in childhood (Table 2). The mean age at LAGH initiation was 38.2 ± 15.6 years. All adult patients were switched from daily GH therapy. No adverse events related to somapacitan have been reported to date. The starting dose of somapacitan was below the manufacturer’s recommendation in 41% of the cases, in keeping with it in 55% of the cases and exceeded the recommended dose in one case (3.4%).

**Table 2:** Characteristics of adult LAGH patients

Patient Characteristics	Adult GHD Patients with LAGH Therapy (*n* = 30)
Age at Diagnosis (years)	20.7 (18.0)
Childhood onset (%)	56.7
Age at initiation of rhGH/LAGH Therapy (years)	24.7 (18.7)
Age at Start of LAGH Therapy (years, range)	38.2 (15.6) (18.3–73.9)
Gender (Male/Female, %)	64.5 / 35.5
Idiopathic/Organic GHD (%)	16.1 / 83.9
“Switch”/First Line Prescription	100.0 / 0.0
Duration of Prior Treatment (years)	13.6 (8.2)
Starting Dose below/equal/above recommended dose (%)	41.4 / 55.2 / 3.4
Percentage Starting Dose to recommended dose (Mean (SD), Median)	86.0 (33.2), 100.0

Values are means (SD), if not otherwise mentioned. In adult patients, no IGF-I or IGFBP3 values were reported

## Discussion

INSIGHTS-GHT is the first study to document the real-world use of all three currently available LAGH agents within the same registry, offering a unique opportunity to assess their utilization and early treatment patterns in routine clinical practice.

Our findings indicate that in the majority of cases the treating pediatric endocrinologists chose a starting dose of LAGH products to be lower than the manufacturers’ recommended starting dose, with a median of 92% of the recommended dose. Several factors may contribute to this conservative dosing approach. Firstly, as an observational study reflecting routine clinical practice, physicians may have opted for cautious dosing to minimize potential adverse effects, particularly those related to transient supraphysiological IGF-I elevation. Secondly, given that 56% of the pediatric patients and all adult patients receiving LAGH were “switch patients” transitioning from daily GH therapy, clinicians may have adjusted dosing based on prior treatment responses rather than strictly adhering to standard initiation doses recommended by the respective manufacturers. Thirdly, there may be a general tendency to individualize therapy based on patient characteristics, such as prepubertal status, body mass index (BMI), or prior growth response. Finally, dosing recommendations for the (LA)GH products are derived from pooled results from US and non-US registration studies, with a tendency to prescribe lower doses in Europe than in the US, reflecting a lower recommended daily dose of rhGH in GHD (25 µg/kg/d in Germany versus 35 µg/kg/d in the US) [10]. While recommended starting doses for patients under the age of 18 years depend on body weight [8], which requires dose rounding in almost all cases, there are four fixed starting doses for adult patients depending on age, previous GH therapy and estrogen therapy. However, in 41% of the adult patients, starting dose was below the manufacturer’s recommendation. The reasons might be similar as for the pediatric patients.

The age at GHD diagnosis and initiation of GH replacement therapy in the pediatric patients of this study was lower than that reported in the PATRO KIDS cohort (7.1 ± 3.6 years vs. 9.8 ± 3.8 years for the latter) [11], and in the large KIGS cohort (10.2 + 4.0 years) [12]. In the INSIGHTS-GHT registry, LAGH therapy is initiated in a relatively young cohort (mean age: 9.2 years) with a majority of prepubertal patients, suggesting that clinicians are integrating LAGH into routine practice at an early stage of treatment. The predominance of idiopathic GHD cases (70%) aligns with previous findings in GH registries, indicating that LAGH is being prescribed across a broad range of GHD etiologies, following a similar pattern of underlying diagnoses.

As this is the first analysis of three LAGH products launched for GH replacement in GHD in Germany within the same study, further follow-up will be essential to assess long-term efficacy, adherence, and safety. Future analyses will also evaluate height velocity, development of IGF-I/IGFBP-3 concentrations in the treatment course, metabolic markers, and adherence patterns in order to determine whether these lower-than-expected starting doses impact on clinical outcomes over time. The inclusion of multiple endocrinology centers across Germany ensures that the study reflects real-world clinical decision-making, providing valuable insights into evolving treatment practices with LAGH.

This interim analysis is subject to inherent limitations of observational studies, including potential selection bias, absence of randomization, and a relatively short follow-up [13]. 

An important unresolved question concerns the trajectory of IGF-I and IGFBP-3 concentrations during follow-up. Measurement of these biomarkers during treatment with LAGH is an optional parameter in the study aimed at assessment of the potential clinical implications of elevated peak and subnormal trough IGF-I concentrations. However, the current follow-up dataset remains limited and does not allow to adequately address this important aspect. Since this is the first report on the use of LAGH in clinical practice - focusing primarily on patient selection and physician preferences - additional longitudinal data are required to adequately address these questions.

In conclusion, the results expand upon previous clinical trial findings by reflecting routine treatment decisions and dose adaptations in both pediatric and adult patients with GHD. These real-world data are particularly valuable in guiding treatment decisions for less common indications for GH therapy, including atypical forms of GHD, where evidence from trials is limited.

## Data Availability

Data sharing is not applicable to this article as datasets are being generated but have not been analysed during the current study.

## Declarations

### Study registration

BfArM Nr. NIS7492, DRKS registry DRKS00027394.

## References

[1] Mameli C, Orso M, Calcaterra V, Wasniewska MG, Aversa T, Granato S, et al. Efficacy, safety, quality of life, adherence and cost-effectiveness of long-acting growth hormone replacement therapy compared to daily growth hormone in children with growth hormone deficiency: A systematic review and meta-analysis. Pharmacol Res. 2023;193:106805. 37236413 10.1016/j.phrs.2023.106805

[2] European Medicines Agency (EMA). Skytrofa (previously Lonapegsomatropin Ascendis Pharma). Summary of Product Characteristics (SmPC). Document EMA/740430/2021 Revision 5 dated 11.01.2022. Internet: https://www.ema.europa.eu/en/medicines/human/EPAR/skytrofa. Accessed on 17.05.2025.

[3] European Medicines Agency (EMA). Sogroya (Somapacitan) Summary of Product Characteristics (SmPC). Document EMEA/H/005030 Revision 2 dated 31.03.2021. Internet: https://www.ema.europa.eu/en/medicines/human/EPAR/sogroya. Accessed on 17.05.2025.

[4] European Medicines Agency (EMA). Ngenla (Somatrogon) Summary of Product Characteristics (SmPC). Document EMEA/H/C/005633 Revision 4 dated 14.02.2022. Internet: https://www.ema.europa.eu/en/medicines/human/EPAR/ngenla. Accessed on 17.05.2025.

[5] Mameli C, Guadagni L, Orso M, Calcaterra V, Wasniewska MG, Aversa T, et al. Epidemiology of growth hormone deficiency in children and adolescents: a systematic review. Endocrine. 2024;85:91–8. 38498128 10.1007/s12020-024-03778-4 PMC11246253

[6] Hoffman AR, Raveendran S, Manjelievskaia J, Komirenko AS, Winer I, et al. Prevalence, treatment patterns, and characteristics of US adults with confirmed or at risk for growth hormone deficiency. Adv Ther. 2025;Online first:22April. 10.1007/s12325-025-03188-6 PMC12085356 40261564

[7] Binder G, Reinehr T, Ibáñez L, Thiele S, Linglart A, Woelfle J, et al. GHD diagnostics in Europe and the US: an audit of National guidelines and practice. Horm Res Paediatr. 2019;92:150–6. 31707392 10.1159/000503783

[8] Maniatis A, Cutfield W, Dattani M, Deal C, Collett-Solberg PF, Horikawa R et al. Long-Acting growth hormone therapy in pediatric growth hormone deficiency: A consensus statement. J Clin Endocrinol Metab. 2024;dgae834. 10.1210/clinem/dgae834 PMC11913077 39672599

[9] Schnabel D, Kreitschmann-Andermahr I, Strasburger CJ, Pittrow D, Pausch C, Woelfle J, et al. Investigating significant health trends in growth hormone treatments registry: rationale, aims and design of a nationwide prospective registry (study protocol). BMC Orphanet J Rare Dis. 2023;18:112. 10.1186/s13023-023-02716-3 PMC10173596 37165422

[10] Backeljauw P, Kanumakala S, Loche S, Schwab KO, Miller BS, Levy R, et al. Safety and effectiveness of Omnitrope^®^ (somatropin) in PATRO children: a multi-center, post-marketing surveillance study comparison of US and international cohort data. Eur J Pediatr. 2022;181:2367–78. 35275291 10.1007/s00431-022-04409-8 PMC9110540

[11] Pfäffle R, Schwab KO, Marginean O, Walczak M, Szalecki M, Schuck E, et al. Design of, and first data from, PATRO children, a multicentre, noninterventional study of the long-term efficacy and safety of Omnitrope(^®^) in children requiring growth hormone treatment. Ther Adv Endocrinol Metab. 2013;4:3–11. 23515245 10.1177/2042018813479644 PMC3593298

[12] Maghnie M, Ranke MB, Geffner ME, Vlachopapadopoulou E, Ibáñez L, Carlsson M, et al. Safety and efficacy of pediatric growth hormone therapy: results from the full KIGS cohort. J Clin Endocrinol Metab. 2022. 10.1210/clinem/dgac517. 36102184 10.1210/clinem/dgac517 PMC9693805

[13] Delgado-Rodríguez M, Llorca J, Bias. J Epidemiol Community Health. 2004;58:635–41. 15252064 10.1136/jech.2003.008466 PMC1732856

